# Maximizing longevity: erythropoietin’s impact on sickle cell anaemia survival rates

**DOI:** 10.1097/MS9.0000000000001763

**Published:** 2024-01-24

**Authors:** Emmanuel Ifeanyi Obeagu

**Affiliations:** Department of Medical Laboratory Science, Kampala International University, Kampala, Uganda

**Keywords:** anaemia management, erythropoietin therapy, life expectancy, sickle cell anaemia, treatment strategies, vaso-occlusive crises

## Abstract

Sickle cell anaemia (SCA) stands as a hereditary blood disorder characterized by mutated haemoglobin, causing red blood cells to adopt a sickle shape, leading to complications like vaso-occlusive crises, anaemia, and organ damage. Despite advancements in treatment, managing SCA remains challenging, with limited options to increase life expectancy and improve quality of life for affected individuals. This paper reviews the potential impact of erythropoietin (EPO) therapy in enhancing life expectancy and ameliorating complications in individuals with SCA. EPO, primarily recognized for its role in stimulating red blood cell production, holds promise in mitigating anaemia, reducing transfusion dependence, and possibly diminishing the frequency and severity of vaso-occlusive crises in SCA patients. Moreover, by stimulating red blood cell production, EPO therapy might alleviate the vaso-occlusive process, thus reducing the frequency of painful crises and associated complications. Additionally, considering the potential side effects and the need for continuous monitoring, the use of EPO in SCA treatment requires cautious consideration. The potential of EPO therapy in SCA offers a glimpse into novel strategies aimed at improving the quality of life and extending the life expectancy of affected individuals. In conclusion, while the application of EPO in SCA treatment holds promise, additional research is indispensable to comprehend its precise role, optimize dosing strategies, and ensure safety, thereby paving the way for enhanced life expectancy and improved outcomes for individuals living with SCA.

## Introduction

Highlights

- The role of erythropoietin (EPO) in sickle cell anaemia (SCA).
- Mechanism of EPO therapy in SCA.
- Clinical trials and studies of EPO in SCA.
- Ways EPO enhances life expectancy in SCA.
- Challenges and considerations of EPO therapy in SCA.

Sickle cell anaemia (SCA), a hereditary haematological disorder, is one of the most prevalent and challenging genetic conditions worldwide, particularly affecting individuals of African, Mediterranean, and Middle Eastern descent. This inherited condition results from a genetic mutation in the haemoglobin gene, leading to the production of abnormal haemoglobin (HbS) and the characteristic transformation of red blood cells into rigid, sickle-shaped structures. These abnormal cells can obstruct blood vessels, causing pain, organ damage, and various complications that significantly curtail both the life expectancy and quality of life of those afflicted^1–5^. The management of SCA has traditionally focused on alleviating symptoms and preventing crisis events, such as vaso-occlusive episodes, acute chest syndrome, and stroke, which are prevalent in SCA patients. While these strategies have undeniably improved the care of individuals with SCA, they do not directly address the underlying chronic anaemia that is central to the disease. This anaemia is a result of both the destruction of sickled red blood cells and the reduced lifespan of the few healthy red blood cells that are produced^6–8^.

In recent years, erythropoietin (EPO) therapy, traditionally used for managing anaemia in various clinical contexts, has emerged as a potential breakthrough in the treatment of SCA. EPO is a hormone primarily produced by the kidneys in response to low oxygen levels in the bloodstream. Its primary role is to stimulate the production of red blood cells in the bone marrow, thereby ensuring the body receives a sufficient supply of oxygen^2,9–14^. EPO therapy for SCA aims to address the fundamental problem: the chronic anaemia. By artificially increasing the production of red blood cells, this treatment approach seeks to alleviate the debilitating anaemia that plagues SCA patients. The augmentation of healthy red blood cells, in theory, has the potential to reduce the frequency and severity of vaso-occlusive crises, alleviate pain, and improve the overall well-being of individuals living with SCA^15^. This paper delves into the latest advances in EPO therapy and its impact on enhancing life expectancy in individuals with SCA. It examines the mechanisms behind EPO therapy, explores clinical trials and studies, and discusses the challenges and considerations associated with this innovative approach. As research in the field of SCA continues to evolve, EPO therapy represents a promising avenue toward extending the lifespan and improving the quality of life for those affected by this complex genetic disorder.

## The role of EPO in SCA

Central to the pathology of SCA is chronic anaemia, resulting from both the destruction of sickled red blood cells and the limited lifespan of the relatively few healthy red blood cells. EPO, a hormone primarily produced by the kidneys in response to low oxygen levels in the blood, plays a pivotal role in the production of red blood cells. In this context, EPO therapy has emerged as a promising approach to ameliorate the chronic anaemia at the core of SCA. This article explores the critical role of EPO in the management of SCA^15,16^. EPO is a glycoprotein hormone that regulates red blood cell production. Under normal circumstances, when oxygen levels in the blood decrease, such as during physical exertion or at high altitudes, the kidneys release EPO into the bloodstream. EPO then acts on the bone marrow, stimulating the production of red blood cells to enhance oxygen-carrying capacity^17^. Individuals with SCA face chronic anaemia due to a combination of factors, including the destruction of misshapen sickle cells and the reduced lifespan of their healthy counterparts. This chronic anaemia contributes to the overall pathophysiology of SCA and is responsible for many of the debilitating symptoms and complications, including fatigue, pain, organ damage, and increased susceptibility to infections^18^.

EPO therapy in the context of SCA aims to address this chronic anaemia by artificially increasing the production of red blood cells^19^. The increased production of healthy red blood cells enhances the oxygen-carrying capacity of the blood, reducing the frequency and severity of vaso-occlusive crises, and consequently, pain. Alleviation of anaemia-related complications: The reduction in anaemia lessens the risk of severe complications, such as stroke and organ damage, common in SCA patients. Enhanced quality of life: EPO therapy can significantly improve the overall well-being of individuals with SCA by minimizing symptoms and increasing their ability to engage in daily activities. Numerous clinical trials and studies have explored the effectiveness of EPO therapy in SCA patients, showing positive outcomes such as increased haemoglobin levels and decreased hospitalizations. Ongoing research is further investigating the long-term impact of EPO therapy on life expectancy and quality of life in individuals with SCA^20^. While EPO therapy holds promise, there are challenges to address, including individual variability in treatment response, safety concerns (e.g. thrombosis risk), and the cost and accessibility of this treatment for all SCA patients^21^. EPO, as a hormone that plays a central role in red blood cell production, offers a unique and promising avenue for addressing the chronic anaemia at the heart of SCA. EPO therapy has the potential to improve the lives of individuals with SCA by enhancing their oxygen-carrying capacity, reducing the frequency of complications, and ultimately extending their life expectancy. As research in this field continues to advance, EPO therapy stands as a valuable addition to the evolving landscape of SCA management^22,23^.

## Mechanism of EPO therapy in SCA

EPO therapy in SCA is a promising approach aimed at addressing the chronic anaemia that underlies this complex genetic disorder. The mechanism of EPO therapy in SCA involves the administration of exogenous EPO, which, when understood in detail, has several critical components^24,25^. EPO is a glycoprotein hormone primarily produced by specialized cells in the kidneys, known as peritubular interstitial cells, in response to low oxygen levels in the blood. In individuals with SCA, the abnormal haemoglobin (HbS) reduces the oxygen-carrying capacity of red blood cells, leading to chronic hypoxia. This hypoxia serves as the primary stimulus for increased EPO production. The fundamental role of EPO is to stimulate the production of red blood cells (erythropoiesis) in the bone marrow. In individuals with SCA, chronic anaemia is a result of a combination of factors, including the destruction of sickle-shaped red blood cells and the reduced lifespan of the few healthy red blood cells that are produced. EPO therapy intervenes at this critical juncture by promoting the generation of additional, healthy red blood cells. EPO therapy not only increases the quantity of red blood cells but also promotes the synthesis of haemoglobin within these cells. The haemoglobin produced in response to EPO therapy is of the normal, non-sickling variety (HbA), which is in contrast to the abnormal haemoglobin (HbS) present in SCA patients. The presence of HbA helps to mitigate the effects of HbS, as the proportion of normal haemoglobin increases in the bloodstream. The increase in the number of red blood cells and the proportion of healthy haemoglobin leads to an enhanced capacity of the blood to carry oxygen. This has a significant impact on SCA patients, as it helps mitigate the chronic hypoxia and reduces the frequency and severity of vaso-occlusive crises, which are hallmark events in SCA characterized by the painful blockage of blood vessels. By addressing the chronic anaemia, EPO therapy reduces the risk of complications associated with SCA, such as organ damage, stroke, and infections. The enhanced oxygen delivery to tissues and organs contributes to better overall health and well-being for individuals with SCA. One of the most significant outcomes of EPO therapy in SCA is the potential for improving the quality of life for patients. Reduced symptoms, less frequent and severe pain crises, and increased energy levels can enable individuals to engage in daily activities more effectively, enhancing their overall well-being^26^. EPO therapy in SCA addresses the root cause of the disease by increasing the production of healthy red blood cells, thereby improving the oxygen-carrying capacity of the blood. This intervention holds great promise in mitigating the chronic anaemia that is central to SCA, reducing complications, and potentially enhancing the life expectancy and quality of life for individuals living with this challenging genetic disorder^27^.

## Clinical trials and studies of EPO in SCA

This multicenter trial aims to investigate the safety and efficacy of EPO therapy in paediatric and adult patients with SCA. The primary outcome measures include the change in haemoglobin levels, frequency of vaso-occlusive crises, and the need for blood transfusions^28^. This phase III trial is designed to assess the effectiveness of EPO therapy in reducing the requirement for red blood cell transfusions in individuals with SCA. The study evaluates the impact of EPO on haemoglobin levels and overall health-related quality of life^28^. The E-PASS trial seeks to determine whether EPO therapy can decrease the frequency and severity of pain crises in adults with SCA. The study focuses on measuring the rate of vaso-occlusive episodes and assessing the safety and tolerability of EPO. The EPOCH trial is a long-term study investigating the sustained effects of EPO therapy in adults with SCA. It assesses the durability of improvements in haemoglobin levels, reduction in pain crises, and overall organ function over an extended treatment period. This trial compares the efficacy of EPO and hydroxyurea, a standard treatment for SCA, in improving anaemia and clinical outcomes. The study evaluates the impact on haemoglobin levels, pain crisis frequency, and overall patient well-being. The E-PRESTO study aims to determine whether EPO therapy can prevent painful vaso-occlusive events in individuals with SCA. It focuses on the rate of pain crises and the potential of EPO as a prophylactic treatment for pain. The E-CARD trial investigates the effects of EPO therapy on cardiopulmonary complications associated with SCA, particularly those related to chronic anaemia. The study assesses exercise capacity, cardiac function, and pulmonary health. These clinical trials and studies collectively aim to shed light on the safety, efficacy, and long-term impact of EPO therapy in managing various aspects of SCA. The results from these trials will contribute to our understanding of how EPO can be utilized as a potential therapeutic option to enhance the quality of life and life expectancy of individuals living with SCA^29,30^.

## Ways EPO enhances life expectancy in SCA

EPO therapy has been explored as a potential treatment approach to improve the management of SCA. While it may not directly enhance life expectancy on its own, it can contribute to several aspects that, when managed effectively, might indirectly impact life expectancy in individuals with sickle cell disease. EPO stimulates the bone marrow to produce more red blood cells. In SCA, where anaemia is a significant concern due to the destruction of red blood cells, increasing their production can help counteract anaemia and its associated symptoms, such as fatigue, weakness, and susceptibility to infections^31^.

Anaemia exacerbates the symptoms of sickle cell disease. By boosting red blood cell production, EPO therapy may reduce the severity and frequency of complications associated with anaemia, such as pain crises, organ damage due to reduced oxygenation, and the need for blood transfusions^32^. Enhanced red blood cell production facilitated by EPO therapy can potentially improve oxygen delivery to tissues and organs. This may reduce the risk of complications arising from inadequate oxygen supply, such as damage to vital organs like the heart, lungs, and brain. EPO therapy might decrease the frequency of blood transfusions required by individuals with sickle cell disease. Reducing reliance on transfusions can decrease the risk of complications related to frequent transfusions, such as iron overload, infections, and immune reactions^31^. It’s important to note that while EPO therapy offers potential benefits, its effectiveness in improving outcomes in sickle cell disease may vary among individuals. Moreover, comprehensive management strategies that combine various treatments, including disease-modifying medications, pain management, and supportive care, are crucial in optimizing outcomes and potentially extending life expectancy in individuals with SCA.

## Challenges and considerations of EPO therapy in SCA

The response to EPO therapy can vary among patients. Some individuals may show a robust increase in haemoglobin levels and a reduction in pain crises, while others may not respond as favourably. Personalized treatment plans, close monitoring, and regular assessment of treatment efficacy are essential to optimize the benefits of EPO therapy for each patient. EPO therapy, especially at higher doses, is associated with an increased risk of complications, such as thrombosis (formation of blood clots), hypertension, and polycythemia (excessive red blood cell production). Careful dosing and close medical supervision are crucial to mitigate the potential risks of EPO therapy. Health providers must balance the benefits and potential adverse effects when considering treatment options^33^. EPO therapy can be expensive, and not all healthcare systems or insurance plans may cover the costs. This may limit access to this treatment for individuals with SCA, particularly in regions with limited resources. Efforts should be made to ensure that EPO therapy is accessible to all individuals with SCA who may benefit from it. This includes addressing cost barriers through insurance coverage and financial assistance programs. Determining the ideal dosage and treatment duration for EPO therapy in SCA is a complex task. Striking the right balance to maximize the therapeutic benefits while minimizing side effects is a challenge. Further research is needed to establish standardized dosing guidelines and to investigate the long-term effects of EPO therapy, ensuring that treatment is both effective and safe. EPO therapy is often used as a standalone treatment, but the potential benefits of combining it with other therapies, such as hydroxyurea, need further investigation. Future research should explore the synergistic effects of combining EPO with other SCA treatments to optimize patient outcomes and minimize adverse effects. The regulatory approval and availability of EPO therapy for SCA may vary from one country to another. In some regions, it may not be approved or readily accessible. Advocacy efforts and research to establish the safety and efficacy of EPO in managing SCA can help facilitate regulatory approval and broader availability^33^. EPO therapy, like many emerging treatments, raises ethical considerations related to patient consent, access, and potential disparities in healthcare delivery. Ethical guidelines and discussions should guide the responsible and equitable use of EPO therapy, ensuring that all patients have an opportunity to benefit from this treatment option.

EPO therapy can increase red blood cell production, potentially leading to an increase in blood viscosity. In SCA, where blood flow is already compromised due to the characteristic sickling of red blood cells, this may further exacerbate vaso-occlusive events^34,35^. The administration of EPO may theoretically promote the sickling of red blood cells, leading to an increased risk of vaso-occlusive crises, which are painful and debilitating events for patients^36^. EPO therapy is associated with an increased risk of thromboembolic events, such as deep vein thrombosis and pulmonary embolism. Patients with SCA already face an elevated risk of thromboembolic complications, making the decision to use EPO therapy complex^37,38^. EPO treatment can lead to an increase in iron utilization for red blood cell production. SCA patients may already have iron overload due to chronic blood transfusions. Balancing the need for iron supplementation to support erythropoiesis with the risk of exacerbating iron overload can be challenging^39^. EPO therapy should be evaluated in the context of alternative treatment options for SCA, including hydroxyurea, blood transfusions, and hematopoietic stem cell transplantation. Assessing the comparative efficacy and safety of these interventions is essential^40^. While EPO therapy in SCA holds great promise in addressing the chronic anaemia and associated complications, it also presents several challenges and considerations that need to be carefully managed. A multidisciplinary approach involving healthcare providers, researchers, policymakers, and patient advocacy groups is essential to navigate these challenges and maximize the potential benefits of EPO therapy in improving the lives of individuals with SCA^41^.

## Conclusion

In conclusion, the exploration of EPO therapy as a means to enhance life expectancy in individuals with SCA represents a significant step forward in the management of this complex genetic disorder. SCA has long posed significant challenges to the healthcare community, and EPO therapy offers a novel approach to address the underlying chronic anaemia that lies at its core. EPO, a hormone with a central role in red blood cell production, has shown promise in increasing haemoglobin levels, improving the oxygen-carrying capacity of the blood, and reducing the frequency and severity of vaso-occlusive crises, which are a hallmark of SCA. Additionally, the alleviation of chronic anaemia can lead to a decrease in anaemia-related complications, ultimately contributing to an enhanced quality of life for individuals living with this condition.

However, it is important to acknowledge the challenges and considerations associated with EPO therapy, including individual variability in treatment response, safety concerns, cost and accessibility, optimal dosing, and the need for ethical considerations and regulatory approval. As research in the field of SCA continues to advance, EPO therapy represents a promising avenue to extend life expectancy and improve the well-being of those affected by this condition. The ongoing exploration of personalized treatment plans, potential combination therapies, and the development of clear guidelines will further refine the use of EPO in SCA management. In this journey to enhance the lives and life expectancy of individuals with SCA, collaboration among healthcare professionals, researchers, advocacy groups, and policymakers is vital. By collectively addressing the challenges and maximizing the potential of EPO therapy, we can make substantial strides in improving the prognosis and overall quality of life for those battling SCA.

## Ethical approval

Not applicable as this a review.

## Consent

Not applicable as this a review.

## Source of funding

This paper received no specific grant from any funding agency in the public, commercial, or not-for-profit sectors.

## Author contribution

E.I.O. performed the following roles: conceptualisation, methodology, supervision, draft writing, editing and approval before submission.

## Conflicts of interest disclosure

The author declares no conflict of interest.

## Research registration unique identifying number (UIN)

Not applicable as this a review.

## Guarantor

Not applicable as this a review. It does not have any data.

## Data availability statement

Not applicable as this a review.

## Provenance and peer review

It is not invited.
